# The difficult problem of the crime of impetus: A proposal for an integrated analysis of complex cases

**DOI:** 10.1192/j.eurpsy.2021.1005

**Published:** 2021-08-13

**Authors:** R. Sperandeo, V. Cioffi, S. Annunziata, B. Muzii, N. Maldonato

**Affiliations:** 1 Phenomena Research Group, Sipgi, Post graduate School of Psychotherapy, Torre Annunziata, Naples, Italy; 2 Department Of Humanistic Studies, University of Naples Federico II, Naples, Italy, Naples, Italy; 3 Neurosciences And Reproductive And Odontostomatological Sciences, University of Naples Federico II, Naples, Italy, Naples, Italy

**Keywords:** Crime of impetus, Forensic psychiatric analysi, Psychopathological analysi, Criminological analysi

## Abstract

**Introduction:**

The evaluation of the crime of impetus poses problems in seeking clinical elements that configure the total or partial defect of mind. Even in the presence of acute psychotic syndromes it is difficult to identify a psychopathological picture that almost overlaps with the times and methods of the crime itself.

**Objectives:**

From a longitudinal perspective, this contribution intends to propose a method of integrating data derived from psychopathological, criminological and forensic psychiatric analyzes, in order to identify the link between them and the criminal act that qualifies or excludes the mental defect.

**Methods:**

In the analysis of a criminal event generated during an acute psychotic episode, it’s described the logic and methodology of integrating criminological, psychopathological and psychiatric forensic data for which it was possible to identify the pathological nature of the event.

**Results:**

The psychopathological analysis allowed the identification of psychotic manifestations before and after the crime. The criminological analysis included the decision to commit the crime, within a framework of alteration of the reality examination and recognized the delusional purpose in the same methods of carrying out the crime. So, the forensic psychiatric analysis clarified the continuity of the psychopathological manifestations in the time frame in which the crime was planned, organized and committed and to codify such manifestations as an acute psychotic episode.

**Conclusions:**

Although it’s not sufficient to circumscribe the criminal act between two pathologically relevant moments, this contribution shows how integrating different methods of analysis makes it possible to identify the quality of the behavior intended as a crime.

